# Comparing the Performance of Raman and Near-Infrared Imaging in the Prediction of the In Vitro Dissolution Profile of Extended-Release Tablets Based on Artificial Neural Networks

**DOI:** 10.3390/ph16091243

**Published:** 2023-09-01

**Authors:** Dorián László Galata, Szilveszter Gergely, Rebeka Nagy, János Slezsák, Ferenc Ronkay, Zsombor Kristóf Nagy, Attila Farkas

**Affiliations:** 1 Department of Organic Chemistry and Technology, Faculty of Chemical Technology and Biotechnology, Budapest University of Technology and Economics, H-1111 Budapest, Hungary; 2 Department of Applied Biotechnology and Food Science, Faculty of Chemical Technology and Biotechnology, Budapest University of Technology and Economics, H-1111 Budapest, Hungary; 3 Department of Innovative Vehicles and Materials, GAMF Faculty of Engineering and Computer Science, John von Neumann University, H-6000 Kecskemét, Hungary

**Keywords:** PAT, Raman imaging, NIR imaging, dissolution profile prediction, convolutional neural network

## Abstract

In this work, the performance of two fast chemical imaging techniques, Raman and near-infrared (NIR) imaging is compared by utilizing these methods to predict the rate of drug release from sustained-release tablets. Sustained release is provided by adding hydroxypropyl methylcellulose (HPMC), as its concentration and particle size determine the dissolution rate of the drug. The chemical images were processed using classical least squares; afterwards, a convolutional neural network was applied to extract information regarding the particle size of HPMC. The chemical images were reduced to an average HPMC concentration and a predicted particle size value; these were used as inputs in an artificial neural network with a single hidden layer to predict the dissolution profile of the tablets. Both NIR and Raman imaging yielded accurate predictions. As the instrumentation of NIR imaging allows faster measurements than Raman imaging, this technique is a better candidate for implementing a real-time technique. The introduction of chemical imaging in the routine quality control of pharmaceutical products would profoundly change quality assurance in the pharmaceutical industry.

## 1. Introduction

The quality of a pharmaceutical solid dosage form is determined by the concentration and the spatial distribution of its components. While the percentage composition of a tablet can be measured with routinely applied chromatographic techniques, characterizing the spatial distribution of the components requires more sophisticated approaches. Vibrational chemical imaging is an excellent tool for this purpose, providing desired information with non-destructive measurements. These methods enable the collection of chemical maps that form hyperspectral data cubes where each spatial point is characterized by a spectrum which carries information about the local composition [1]. In past decades, instrumentation did not allow fast measurements. Therefore, acquiring a usual chemical map often required a whole day, making exploiting its real potential in quality assurance impossible. In this state, chemical imaging could only be used for R&D purposes. However, nowadays, state-of-the-art techniques can record chemical maps in a few minutes [2]. This drastic increase in throughput might result in at-line or even in-line applications.

In the analysis of pharmaceutical tablets, two different types of vibrational chemical imaging techniques are commonly utilized: Raman [3] and near-infrared (NIR) [4] imaging. Raman spectroscopy relies on the occurrence of inelastic light scattering; this means that, when a photon collides with a molecule, its energy increases or decreases. The quantity of photons with certain energy changes depends on the structure of the analyzed molecules; therefore, Raman spectra carry information about the sample composition [5]. Raman imaging of tablets used to be a slow process, where at least a minute of exposition was necessary to obtain a single spectrum [6]. However, appliances developed in the past few years can record thousands of spectra in one minute [7] and new approaches like stimulated Raman scattering are even faster [8]; thus, nowadays a large number of tablets can be analyzed with this technique in a reasonable time. Generally, Raman spectroscopy is known for its sensitivity to ambient light; the analysis of materials prone to fluorescence tends to be difficult; however, it has the advantage of good spectral and spatial resolution [9]. NIR spectroscopy is based on light absorption; photons in the NIR range can be absorbed by molecules to excite the combinations or overtones of molecular vibrations. NIR imaging has become an excellent candidate for pharmaceutical quality analysis as, after recent developments, its measurement speed enables even in-line applications [10]. Moreover, quantum cascade laser technology can further improve the already impressive measurement speed [11]. When compared to Raman spectroscopy, NIR spectroscopy has a lower spatial resolution and, due to the broad bands of the spectra, it might be more difficult to differentiate between components; however, it is less sensitive to ambient light.

In the past 15 years, both NIR and Raman imaging have been used in the analysis of pharmaceutical tablets to measure their active ingredient content [12,13], to characterize the size of components [14,15], to measure the coating thickness of film-coated tablets [16,17], to characterize the distribution of polymorphic forms of a drug [18,19], to detect changes after storage under stress [20,21], to recognize counterfeit products [22,23], to identify the components of a formulation [24] and to predict the dissolution profile of tablets [6,25,26]. It is evident that both techniques are capable of great things; however, the aforementioned works provide little help in deciding which imaging method is worth using to achieve a specific goal. There are only a few works in the literature where the authors directly compare Raman and NIR imaging by using them to perform the same measurement. The works of Šašić [27,28,29], Carruthers et al. [30] and Lopes et al. [31] analyze Raman and NIR maps of a handful of tablets in great detail. It was found that the two techniques yield generally similar results, although, in distribution maps obtained by Raman imaging, the boundaries of particles are clearer and Raman imaging is a better choice for components with low concentrations. Mitsutake et al. [24] compared the capability of Raman and NIR imaging in the analysis of solid dispersions; Raman was more sensitive to the active ingredient, while NIR was more sensitive to the excipient. However, to our knowledge, there are no works in the literature where Raman and NIR imaging are compared using several hundred samples. With such a dataset, a more quantitative comparison of the techniques can be performed; thus, a more informed choice can be made between these two spectroscopic methods.

Although nowadays a formulation scientist can choose from a plethora of sophisticated drug delivery designs [32,33], the most frequently manufactured sustained-release products rely on more simple principles [34]. The relationship between the manufacturing properties of such a tablet and its dissolution profile is described by an unknown function. When a sufficient amount of data is available, various machine learning techniques can be utilized to estimate this function. Artificial neural networks (ANNs) are the most popular in this field, as they have proved their ability many times to model basically any kind of phenomenon [35]. Recently, ANNs have been effectively employed to predict the dissolution profile of tablets using data derived from spectroscopic measurements or other sources related to their composition [36]. Furthermore, due to the rapid development of image processing with convolutional neural networks (CNNs), it is now feasible to create models that consider the spatial information carried by images [26,37]. Hence, when aiming to predict the dissolution profile of a tablet based on its chemical image, CNNs can be applied to extract complex information about the tablet composition. In our earlier studies [6,26], we applied Raman imaging and used CNNs to process the obtained images to predict the dissolution profile of tablets. However, it remains uncertain whether NIR imaging is better suited for this purpose, as it offers advantages such as faster measurements and lower sensitivity to ambient light.

Consequently, our article has two main goals. Our first goal is to determine whether NIR or Raman imaging is the better choice for characterizing the properties of hydroxypropyl methylcellulose (HPMC) in extended-release tablets with the intention of using the acquired data to predict the rate of drug release from these tablets. Secondly, we intend to exploit the capability of CNNs to extract information about the dissolution directly from the chemical images to obtain a more thorough characterization of the samples. By combining fast chemical imaging and the processing ability of CNNs, we are at the brink of a new era in the quality assurance of pharmaceutical products.

## 2. Results and Discussion

### 2.1. Evaluation of the Chemical Images

**Figure 1:**
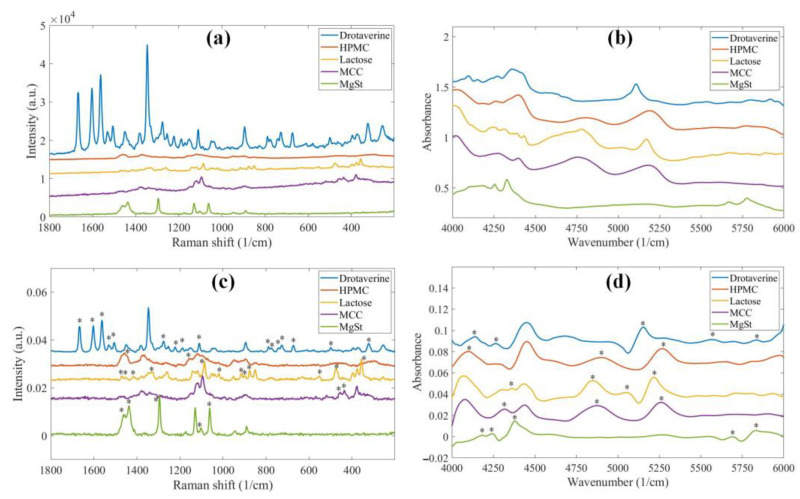
(**a**) Raw Raman spectra; (**b**) raw NIR spectra; (**c**) Raman spectra after preprocessing (smoothing, baseline correction and normalization); (**d**) NIR spectra after preprocessing (1st derivative) of the pure components of the sustained-release formulation. The asterisks mark peaks characteristic to the components after preprocessing.

The raw and preprocessed pure component spectra of the sustained-release tablets are shown in Figure 1. In Raman spectroscopy, drotaverine hydrochloride (DR) has solid signals compared to the excipients; however, its intensity becomes comparable to theirs after normalization. Generally, the components are more distinguishable in the Raman spectra, as all of them have several characteristic peaks. The NIR spectra appear more similar at first glance, as the typical broader peaks of the compounds strongly overlap. The first derivative enhances the differences; however, HPMC and microcrystalline cellulose (MCC) are still very similar. The ability to distinguish between HPMC and MCC is crucial, because the concentration of HPMC in the tablets and its particle size can only be determined accurately if it is not confused with other components.

**Figure 2:**
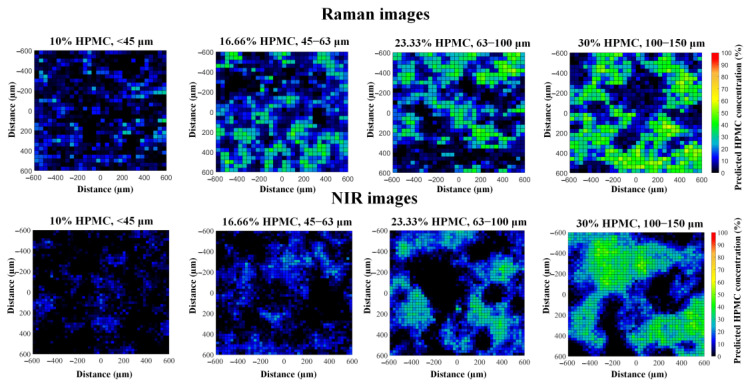
Raman and NIR images of the HPMC distribution of sustained-release tablets with various HPMC concentrations and particle sizes.

The effectiveness of the recognition of HPMC can be evaluated by inspecting the concentration maps of HPMC obtained with the classical least squares (CLS) method (Figure 2). In general, the concentration maps of both imaging methods have characteristics that correlate well with the known properties of the tablets. At higher HPMC concentrations, the number of green pixels increases; these mark regions rich in HPMC. By comparing the size of the green/blue regions, it can also be observed that the concentration maps give a good representation of the particle size of HPMC. Small particle sizes result in a more homogeneous distribution, while combinations of high-intensity regions and empty spaces indicate large particle fractions. This visual analysis implies that both imaging methods were able to successfully differentiate between HPMC and MCC, as the latter has the same concentration and particle size in all tablets; thus, the differences seen must be caused by HPMC.

In the case of this formulation, the quality of the Raman spectra was strongly influenced by the significant fluorescence caused mainly by MCC. As a result, the fluorescent baseline dominated the spectra, making analysis more difficult. Although the components could still be differentiated, this implies that, in some scenarios, Raman imaging might not be applicable due to fluorescent effects; thus, it is vital to have alternative techniques at hand.

### 2.2. Prediction of the Dissolution Profiles

**Figure 3:**
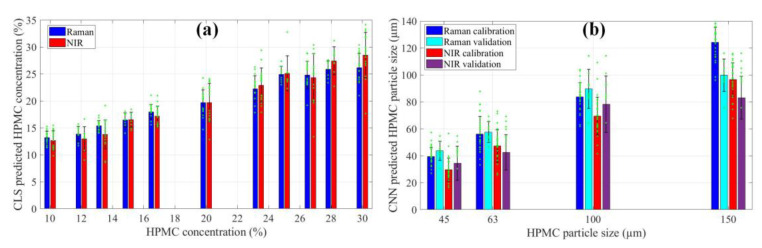
(**a**) CLS predictions of the concentration of HPMC; (**b**) HPMC particle size predicted with CNN. Green asterisks represent individual predicted values, black error bars show the standard deviation.

The capability of Raman and NIR imaging can also be quantitatively compared by evaluating the predictions of HPMC concentration and particle size (Figure 3). Generally, the CLS method tends to overestimate the concentration of compounds with a more intense signal and underestimate the other components. In this scenario, both spectroscopic techniques underestimated the concentration. To ensure better comparability of results, the difference between the averages of the real and predicted concentrations was added to the data before plotting (this difference was 6.85 for Raman and 14.72 for NIR). In Figure 3a, it is evident that the predictions of both Raman and NIR imaging follow the trend of increasing concentration, but NIR imaging does so with a higher standard deviation. As for the predictions of particle size by CNN (Figure 3b), they have a similar range, but NIR consistently predicts smaller sizes. It can also be observed that, in the case of the 100–150 µm fraction (the columns at 150 on Figure 3b), the validation samples are predicted with noticeably lower values with both techniques. Most likely there was not enough training data for the CNN to appropriately learn to quantify these larger particles. Consequently, the drug release rate from these tablets was later underestimated by the dissolution prediction model.

**Figure 4:**
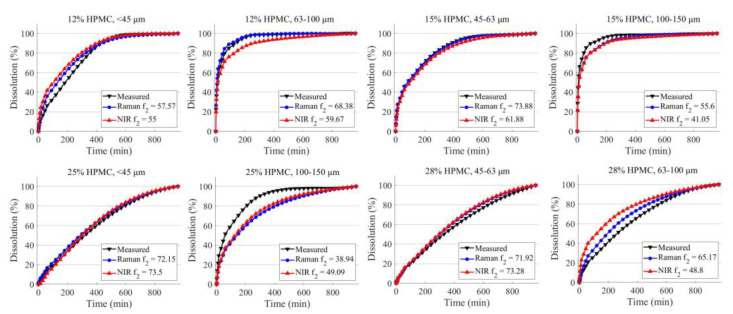
The average of the predicted dissolution profiles of the validation tablets obtained based on Raman and NIR imaging.

The final assessment of the two imaging techniques involves predicting the dissolution profile based on the HPMC concentration and particle size derived from the chemical maps (Figure 4). Both techniques managed to produce sufficient information for the ANNs, as the shape of the dissolution profiles closely resembles the measured dissolution in all cases. The average f_2_ is 62.7 for Raman and 57.8 for NIR. Predictions based on Raman imaging had an average root mean squared error (RMSE) value of 4.72 and a coefficient of determination (R^2^) value of 0.95, while NIR imaging yielded an RMSE of 6.44 and an R^2^ of 0.93. Raman imaging exhibited more accurate predictions in six out of the eight cases. However, the most significant deviations were observed in formulations with high HPMC concentration and a larger particle size fraction (25% HPMC 100–150 μm particle size and 28% HPMC 63–100 μm particle size). This less optimal performance could likely be attributed to the ANNs not having sufficient examples where high HPMC concentration is offset by the large particle size, thus hindering the ability to predict the dissolution rate accurately.

## 3. Materials and Methods

### 3.1. Materials

The model active ingredient of the tablets, drotaverine hydrochloride (DR) was purchased from Sigma Aldrich (Munich, Germany). The binder of the tablets was microcrystalline cellulose (MCC) with the brand name Vivapur 200, supplied by JRS Pharma (Rosenberg, Germany). As filler, α-lactose monohydrate was utilized; the supplier of type Granulac^®^ 70 was Meggle Pharma (Wasserburg, Germany). The component responsible for sustained release was hydroxypropyl methylcellulose (HPMC); specifically, its type was K100M DC2; this ingredient was a gift from Colorcon (Budapest, Hungary). Lastly, the lubricant of the tablets was magnesium stearate; this compound was obtained from Hungaropharma Ltd. (Budapest, Hungary). For the preparation of the dissolution medium, a 37% hydrochloric acid (HCl) solution was acquired from Merck (Darmstadt, Germany).

### 3.2. Methods

#### 3.2.1. Sieving of Components

For the sieving of HPMC, four sieves were used; their sizes pore were 45 µm, 63 µm, 100 µm and 150 µm. Consequently, four different particle size fractions were obtained. The sieves were subjected to a 2 mm amplitude vibration by a CISA BA 200N (Barcelona, Spain) sieve shaker; the vibration was applied until no more changes could be observed in the mass of the sieve fractions. The following fractions were collected in the case of HPMC: less than 45 µm (<45 µm), between 45 and 63 µm 45–63 µm), between 63 and 100 µm (63–100 µm), and between 100 and 150 µm (100–150 µm).

#### 3.2.2. Preparation of Tablets

A single punch tablet press manufactured by Dott Bonapace, model CPR-6 (Limbiate, Italy) was utilized to prepare tablets. The formulation is a sustained-release tablet which uses DR as the drug, HPMC as the matrix polymer, MCC as the binder, lactose as the filler and MgSt as the lubricant. This formulation was compressed with 14 mm concave punches at a compression force of 15 kN with a target weight of 500 mg. These tablets were compressed into 36 different compositions, where the concentration and particle size of HPMC were varied. Table 1 shows the composition of these tablets. Five tablets were prepared for each composition; four were used for the dissolution testing and the remaining ones for the characterization of the formulation.

The tablets were characterized by measuring their individual weight, height, crushing strength and friability using 10 tablets. The results are shown in Table 2. Additionally, digital images of the tablets are presented in the Supplementary Materials (Figure S1).

#### 3.2.3. Fast Raman Imaging

A Thermo Scientific DXR3xi (Waltham, MA, USA) appliance was used to perform Raman imaging of the surface of tablets. A 785 nm laser was used with 30 mW power for excitation and a microscope objective with 20× magnification was utilized to focus the beam on the surface of the tablets, which were moved with an automated sample stage. The Raman spectra were acquired between a Raman shift of 200 and 1800 cm^−1^. Table 3 shows the measurement settings. The size of the mapped region was chosen in a way that the map should contain several particles even when the largest size fraction is used.

#### 3.2.4. Near-Infrared Chemical Imaging

Reference spectra of the pure components were collected using a Spectrum 400 FT-IR/FT-NIR apparatus manufactured by Perkin Elmer (Waltham, MA, USA) with a near-infrared reflectance accessory (NIRA) which contains InGaAs detector. The NIR reference spectra were recorded in the wavenumber range of 10,000–4000 cm^−1^, the resolution was 16 cm^−1^, the data interval was 2 cm^−1^ and 32 scans were accumulated. Chemical imaging of the tablets was carried out using a Perkin Elmer Spotlight 400 FT-IR/FT-NIR Image System (Perkin Elmer, Waltham, MA, USA) which contains 2 × 8 MCT detectors for image mode. The spectra of the images were acquired between the wavenumbers of 7800 and 4000 cm^−1^, the resolution was 16 cm^−1^, the data interval was 8 cm^−1^ and 2 scans were accumulated at each point. The further settings used during the imaging of the tablets are shown in Table 3.

#### 3.2.5. In Vitro Dissolution Testing

In order to record the in vitro dissolution profile of the tablets, a Hanson SR8-Plus apparatus (Chatsworth, CA, USA) was utilized, using the paddle method (USP II). The tablets were placed in vessels containing 900 mL of HCl solution, the pH value was set to 1.2, the solution was stirred with a paddle speed of 100 rpm and the temperature was 37 ± 0.5 °C. The concentration of DR in the dissolution medium was measured by taking samples with an automatic syringe pump (a Hanson Autoplus 8 Maximizer, Chatsworth, CA, USA) through filters with a pore size of 10 µm. The samples were taken at the following time points: 2, 5, 10, 15, 30, 45 and 60 min, then each 30 min until 960 min. In order to determine the concentration of the drug in the dissolution medium, the collected samples were passed through 10 mm flow through cuvettes, where their absorbance was measured at 302 nm with an UV-VIS spectrophotometer (Agilent 8453, Hewlett-Packard, Palo Alto, CA, USA).

### 3.3. Data Processing

Mathematical processing of the acquired data was realized using version 9.8 of MATLAB with Deep Learning Toolbox 14.0 (Mathworks, Natick, MA, USA) and with PLS_Toolbox 8.8.1 (Eigenvector Research, Manson, WA, USA).

#### 3.3.1. Processing of Chemical Images

The classical least squares (CLS) technique was implemented to convert the Raman and NIR images to concentration maps of HPMC. This required the acquisition of the spectra of the pure constituents of the tablets with both spectroscopic techniques; CLS uses these to predict the concentration of the components in the points of the chemical image. Before applying CLS, the spectra received the following preprocessing: smoothing (Savitzky-Golay, 2nd order polynomial, 15 window width), baseline correction (Automatic Whittaker filter, lambda = 10,000, *p* = 0.001), and normalization for Raman spectra and 1st derivative (Savitzky-Golay, 2nd order polynomial, 15 window width) for NIR spectra. The pure component spectra were treated the same way. As a result, the CLS method predicted a 31×31 (Raman) or 48×48 (NIR) concentration map of HPMC per tablet.

**Figure 5:**
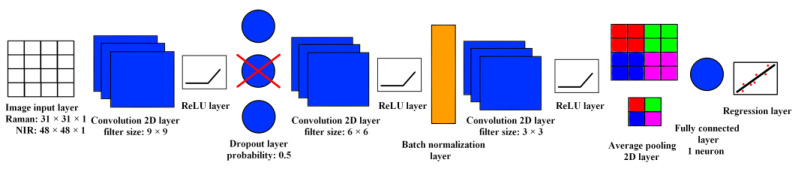
The structure of the CNN used for the prediction of the particle size of HPMC.

Two types of information were extracted from these HPMC concentration maps. Firstly, the HPMC concentration of the tablet was characterized by averaging the HPMC concentration values for all the points of the map. Secondly, the particle size of HPMC was predicted using a CNN. The architecture of this CNN is shown in Figure 5.

The CNN’s first layer is an image input layer; this needs to have the same dimensions as the image to be processed. The recognition of the features in the image happens in the convolution 2D layers. With the application of filters that are basically a small matrix of numbers, the layer performs the mathematical operation of convolution. When this convolution is performed on a certain pixel, the value of the pixel changes based on the values of the other pixels surrounding it. These filters slide through the whole image row by row; as a result, we obtain an image where the features belonging to that filter are highlighted. When the neural network is trained, the values inside the filters are tuned to recognize the features that are present in the training dataset. These features can be simple edges or textures, but also more complex shapes or even whole objects. In the case of our dataset, the CNN needs to learn to recognize the particles of HPMC occurring in different sizes and color intensities. The convolution layers are followed by rectified linear unit (ReLU) layers. These perform a more simple task; they change the values coming out of the previous layer according to a certain function. The ReLU function changes negative values to 0, while positive values are not changed. A transfer function like the ReLU is required to make the training process of the neural network more reliable. A dropout layer is included in the CNN to help prevent overfitting, which is a common problem of machine learning. This layer adds regularization to the training of the neural network by randomly deactivating some of the neurons; in this way it is less likely that the CNN learns the exact pattern of the training data, resulting in poor generalization capability. The batch normalization layer makes the training process more effective by normalizing the incoming data (shifting to a mean of 0 and standard deviation of 1). The average pooling 2D layer reduces the spatial dimensions of the image while the important features are retained; in this way the training process becomes less computationally demanding. The fully connected layer and regression layer are responsible for finding the correlation between the features found by the convolution layers and the response variable. In this case, the detected edges and shapes must be used to calculate the particle size of HPMC. More precisely, as a target value we chose the pore size of the sieve with the smallest pores that the HPMC could pass through.

This CNN was trained by using the maps of 96 of the 112 calibration tablets as training and 16 as validation. The training was executed using the Adam training function; validation was performed after every 5 epochs, learning rate was initially 0.001, the drop period of the learning rate was 10 epochs, the learning rate drop factor was 0.9 and the maximum number of epochs was 100. After the training was completed, the HPMC particle size of the tablets belonging to the 8 validation settings was predicted.

#### 3.3.2. Creation of Model for Prediction of Dissolution Profiles

The dissolution profiles were predicted using a simple feedforward backpropagation ANN with one hidden layer. The ANNs were designed with two inputs: the predicted HPMC concentration and the particle size of HPMC provided by the CNN model. In order to find the ideal neuron number in the hidden layer, training was performed with 1–10 hidden layer neurons, each utilizing the tangent sigmoid transfer function. The last layer of the neural network was the output layer consisting of 37 neurons with linear transfer functions; each neuron predicted one point of the dissolution curve. The ANNs were trained using the Bayesian regularization algorithm, and the training was run until 1000 epochs were reached or, as an alternative stopping criterion, when the gradient of the performance function (mean squared error) became less than 10^−7^. For each hidden layer neuron number, 100 training runs were performed and the best models were selected by using these models to predict the dissolution profiles of the validation formulations. The selection criterion was based on calculating the f_2_ similarity factor of the predictions. The f_2_ value (Equation (1)) was specifically designed to compare dissolution profiles. It was calculated in such a way that the points of the dissolution curve where dissolution had exceeded 85% were discarded (except the first point after 85%). In this way, only the meaningful part of the curves is evaluated.

(1) $$f_{2}=50\log_{10}\left\{{\left[1+\frac{1}{n}\overset{n}{\underset{i=1}{\sum }}w_{t}{\left(R_{t}-T_{t}\right)}^{2}\right]}^{0.5}\times 100\right\}$$

In this equation, n stands for the number of sampling points in the dissolution curve, w_t_ is an optional weighing factor and R_t_ is the dissolution percentage measured at time point t, while T_t_ is the predicted dissolution percentage at time point t.

The difference between the measured and predicted dissolution profiles was also characterized using the root mean squared error (RMSE) value (Equation (2)).

(2) $$\mathrm{RMSE}=\sqrt{\frac{\sum_{i=1}^{n}{\left(R_{t}-T_{t}\right)}^{2}}{n}}$$

The coefficient of determination (R^2^) was also utilized to describe the accuracy of the predictions (Equation (3)).

(3) $$R^{2}=1-\frac{\sum_{i=1}^{n}{\left(T_{t}-R_{t}\right)}^{2}\;}{\sum_{i=1}^{n}{\left(T_{t}-\overset{-}{R}\right)}^{2}}$$

In Equations (2) and (3) the symbols have the same meaning as in Equation (1), and $\overset{-}{R}$ is the average of measured values.

## 4. Conclusions

The advent of fast chemical imaging techniques might mark the dawn of a new era in industrial quality analysis. State-of-the-art equipment now allows measurements that previously took an entire day a decade ago to be performed in just a few minutes. In the pharmaceutical industry, both NIR and Raman imaging demonstrate great potential. This study compared these two techniques in characterizing sustained-release tablets, where dissolution rate was determined by the concentration and the particle size of the HPMC, the matrix polymer. The NIR instrument enabled approximately seven times faster measurements and, after applying CLS to the hyperspectral images, it was found that both techniques could efficiently characterize the properties of HPMC. The dissolution profile was predicted with good accuracy by both NIR and Raman imaging, with Raman being slightly more accurate. However, the greater speed of NIR imaging makes it the preferred choice in most scenarios over Raman imaging.

With the proposed technique, the quality of manufactured tablets can be evaluated in great detail and the future possibility of in-line application of chemical techniques would enable 100% of the prepared products to be characterized. This would bring about a revolutionary change in pharmaceutical quality assurance, eliminating the risk of administering tablets of inadequate composition to patients. Consequently, the safety of pharmaceutical products would be unequivocally assured.

## Figures and Tables

**Table 1:** The concentration of components and particle size of HPMC in the tablets used to calibrate and validate the dissolution prediction models.

Name of Formulation	DR Concentration (*w*/*w*%)	HPMC Concentration (*w*/*w*%)	MCC Concentration (*w*/*w*%)	Lactose Concentration (*w*/*w*%)	MgSt Concentration (*w*/*w*%)	HPMC Size Fraction
Tablets used for calibration						
DR01	8	10	20	60	2	<45 µm
DR02	8	10	20	60	2	45–63 µm
DR03	8	10	20	60	2	63–100 µm
DR04	8	10	20	60	2	100–150 µm
DR05	8	13.33	20	56.67	2	<45 µm
DR06	8	13.33	20	56.67	2	45–63 µm
DR07	8	13.33	20	56.67	2	63–100 µm
DR08	8	13.33	20	56.67	2	100–150 µm
DR09	8	16.66	20	53.34	2	<45 µm
DR10	8	16.66	20	53.34	2	45–63 µm
DR11	8	16.66	20	53.34	2	63–100 µm
DR12	8	16.66	20	53.34	2	100–150 µm
DR13	8	20	20	50	2	<45 µm
DR14	8	20	20	50	2	45–63 µm
DR15	8	20	20	50	2	63–100 µm
DR16	8	20	20	50	2	100–150 µm
DR17	8	23.33	20	46.67	2	<45 µm
DR18	8	23.33	20	46.67	2	45–63 µm
DR19	8	23.33	20	46.67	2	63–100 µm
DR20	8	23.33	20	46.67	2	100–150 µm
DR21	8	26.66	20	43.34	2	<45 µm
DR22	8	26.66	20	43.34	2	45–63 µm
DR23	8	26.66	20	43.34	2	63–100 µm
DR24	8	26.66	20	43.34	2	100–150 µm
DR25	8	30	20	40	2	<45 µm
DR26	8	30	20	40	2	45–63 µm
DR27	8	30	20	40	2	63–100 µm
DR28	8	30	20	40	2	100–150 µm
Tablets used for validation						
DRV01	8	12	20	58	2	<45 µm
DRV02	8	12	20	58	2	63–100 µm
DRV03	8	15	20	55	2	45–63 µm
DRV04	8	15	20	55	2	100–150 µm
DRV05	8	25	20	45	2	<45 µm
DRV06	8	25	20	45	2	100–150 µm
DRV07	8	28	20	42	2	45–63 µm
DRV08	8	28	20	42	2	63–100 µm

**Table 2:** Basic characterization of the prepared tablets.

Property	Value
Weight	503.8 ± 2.8 mg
Height	3.77 ± 0.07 mm
Crushing strength	98.3 ± 9.3 N
Friability	0.79%

**Table 3:** Comparison of Raman and NIR imaging settings.

	Raman Imaging	NIR Imaging
Mapped area size	1200 × 1200 µm^2^	1200 × 1200 µm^2^
Step size	40 µm	25 µm
Number of points	31 × 31	48 × 48
Spectrum measurement time	0.1 s	0.014 s
Number of scans	3	2
Map measurement time	5.8 min	1.1 min

## Data Availability

Data is contained within the article.

## Supplementary Materials

The following supporting information can be downloaded at: https://www.mdpi.com/article/10.3390/ph16091243/s1, Figure S1. Images of the tablets acquired with a digital camera.
