# Supplementary figures and images for: Comparing the Performance of Raman and Near-Infrared Imaging in the Prediction of the In Vitro Dissolution Profile of Extended-Release Tablets Based on Artificial Neural Networks
Source: Pharmaceuticals (Basel). 2023 Sep 1;16(9):1243. doi: 10.3390/ph16091243 (PMC10534500; doi:10.3390/ph16091243)

Figure S1. Images of the tablets acquired with a digital camera.

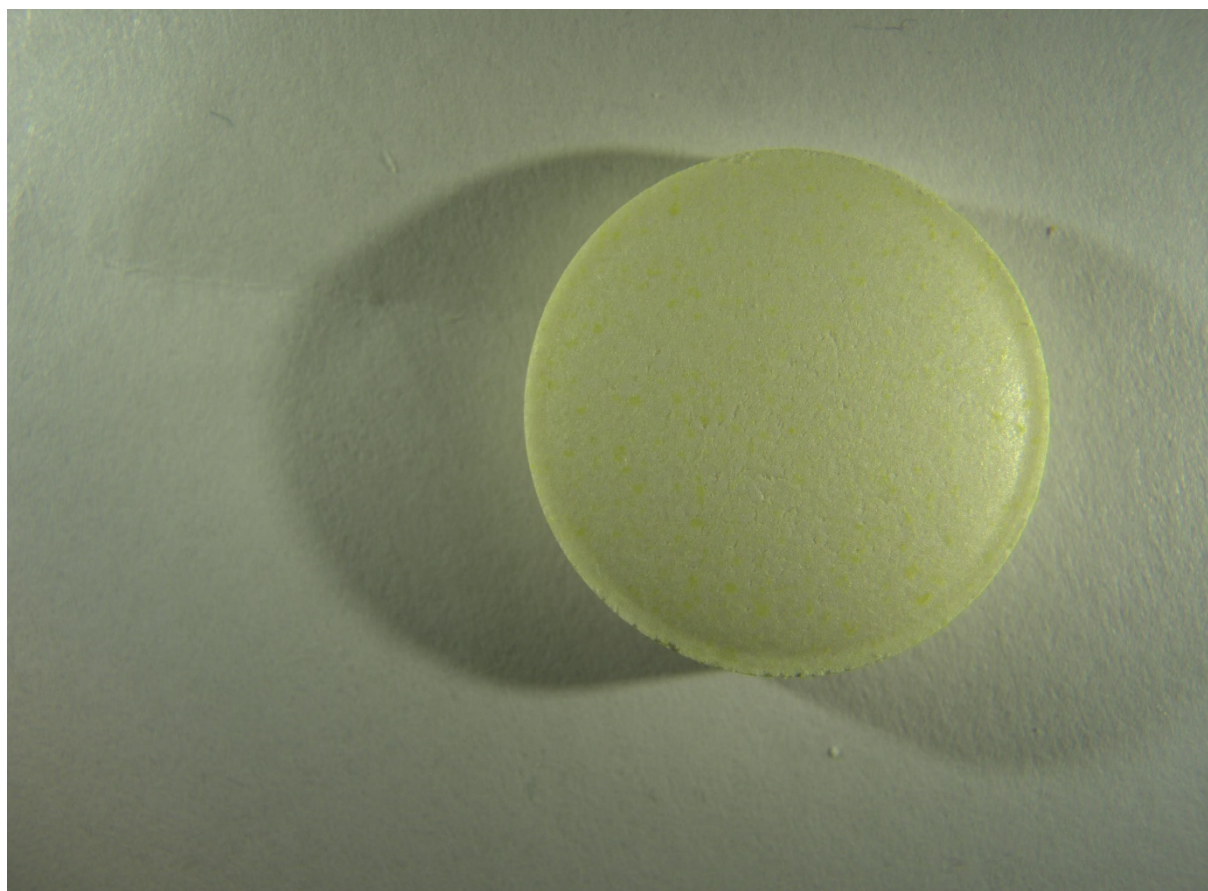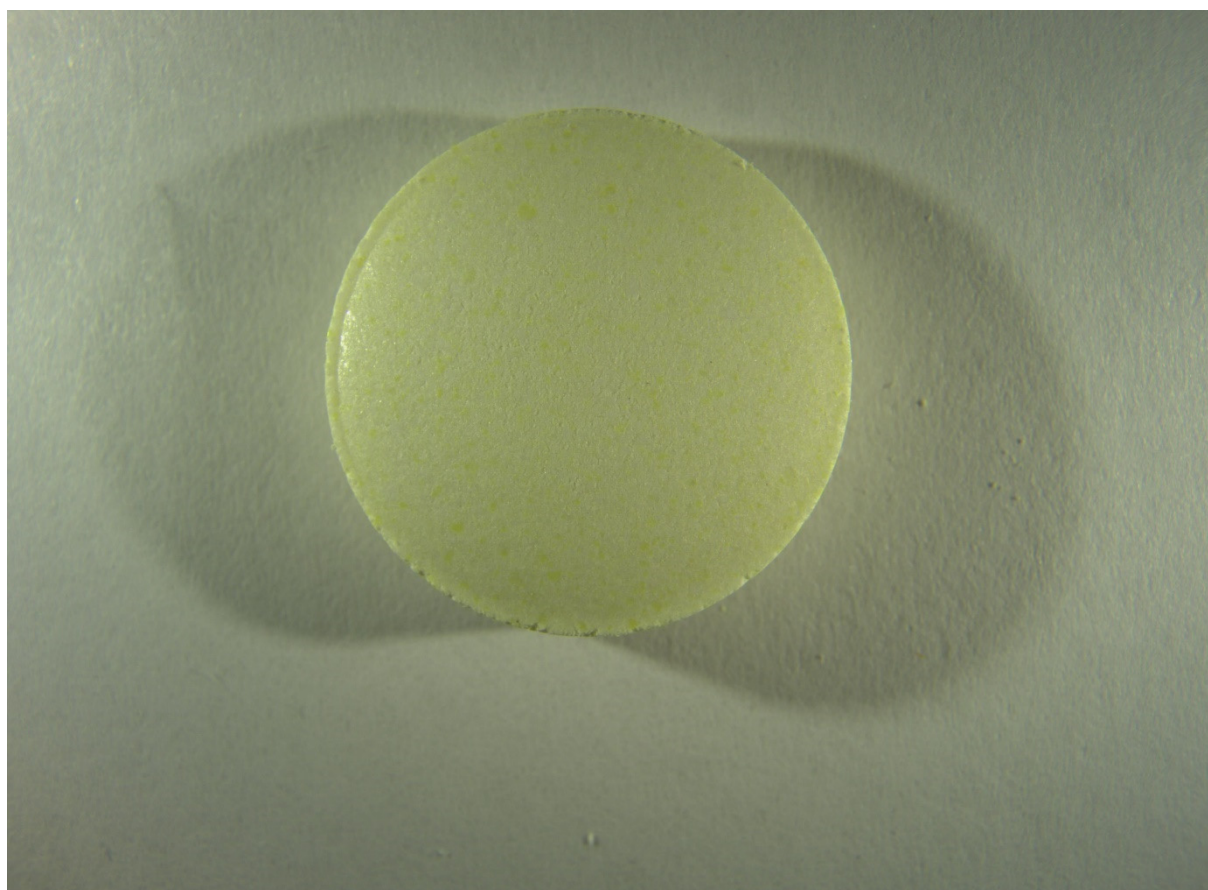

Supplement: Supplementary file 1 [file pharmaceuticals-16-01243-s001.zip › pharmaceuticals-2576626-supplementary.pdf]
